# M1 macrophages polarized by crude polysaccharides isolated from *Auricularia polytricha* exhibit anti-tumor effect on human breast cancer cells

**DOI:** 10.1038/s41598-024-58208-2

**Published:** 2024-04-08

**Authors:** Sunita Nilkhet, Kuljira Mongkolpobsin, Chanin Sillapachaiyaporn, Nichaporn Wongsirojkul, Tewin Tencomnao, Siriporn Chuchawankul

**Affiliations:** 1 Program in Clinical Biochemistry and Molecular Medicine, Department of Clinical Chemistry, Faculty of Allied Health Sciences, Chulalongkorn University, Bangkok, 10330 Thailand; 2 Department of Transfusion Medicine and Clinical Microbiology, Faculty of Allied Health Sciences, Chulalongkorn University, Bangkok, 10330 Thailand; 3 Department of Clinical Chemistry, Faculty of Allied Health Sciences, Chulalongkorn University, Bangkok, 10330 Thailand; 4 Immunomodulation of Natural Products Research Unit, Chulalongkorn University, Bangkok, 10330 Thailand

**Keywords:** Cancer, Immunology, Molecular medicine

## Abstract

Breast cancer has been reported to correlate with the infiltration of tumor-associated macrophages (TAMs) or M2-like macrophages in tumor microenvironment (TME) that could promote breast cancer progression. In contrast, M1-like macrophages displayed anti-tumor activity toward cancer. This study was focused on *Auricularia polytricha* (AP), a cloud ear mushroom, which has been reported for anti-tumor activity and immunomodulation. AP extracts were screened on differentiated THP-1 macrophages (M0). Results demonstrated that water extract (APW) and crude polysaccharides (APW-CP) could upregulate M1-related genes and cytokines production (IL-6, IL-1 β and TNF-α) significantly. Moreover, APW and APW-CP showed a high expression of CD86 (M1 marker) compared to M0. The NF-κB signaling pathway is crucial for pro-inflammatory gene regulation. The APW and APW-CP treatment showed the induction of the NF-κB pathway in a dose-dependent manner, which related to the β-glucan content in the extracts. Furthermore, APW-CP polarized macrophages were investigated for anti-tumor activity on human breast cancer cells (MCF-7 and MDA-MB-231). Results showed that APW-CP could inhibit the invasion of breast cancer cells and induce apoptosis. Therefore, M1 macrophages polarized by APW-CP showed anti-tumor activity against the breast cancer cells and β-glucan may be the potential M1-phenotype inducer.

## Introduction

Breast cancer shows the highest estimated incidence in women (30%) and is ranked the second cause of cancer deaths after lung cancer^1^. With the intervention combinatory therapeutic techniques, the relapse has been considerably reduced, though there may be an incidence of side effects, including cardiotoxicity, lymphedema and pneumonitis^2^. However, the presence of multiple subtypes and molecular patterns such as, HER2-negative, HER2-positive, and triple-negative breast cancer (TNBC) challenges for effective treatment and propels the requirement for personalized medicine for the patients. Tumor microenvironment (TME) correlated with tumor progression, invasion, and poor prognosis^3^. Generally, TME is a network of immune cells, stromal cells, and extracellular matrix (ECM) that balances the homeostasis at a tumor site. However, the advanced stage of the disease can turn surrounding cells in TME into protumorigenesis phenotypes to support their development^4^. Tumor-associated macrophages (TAMs) are dominant immune cells presented in TME with high plasticity. Two distinct phenotypes of macrophages are found; classical activated macrophages (M1) and alternatively activated macrophages (M2). TAMs share similarity to the M2-like phenotype by expressing surface markers such as CD206 and CD163.

In addition, M2 macrophages regulate the production of IL-10, TGF-β, chemokines, growth factors, and matrix metalloproteinases (MMPs), which induce angiogenesis and metastasis of tumors^5^. Interestingly, previous studies reported the capability of several compounds that exhibited macrophage repolarization from M2 to M1 phenotypes and suppression of tumorigenesis. For example, mushroom extracts such as *Antrodia camphorate*^6^, Huaier extract^7^, and Agarikon^8^ have been shown to induce macrophage repolarization to M1 by suppressing the M2 phenotype, and inhibition of tumor growth. *Auricularia polytricha* (AP), or cloud ear mushroom, has been shown to be a promising mushroom for cancer treatment, with anti-tumor activity^9^, anti-inflammation^10^, anti-oxidation^11^, and immunomodulation^12^. However, the effect of AP on macrophage polarization and its anti-tumor property has never been reported.Thus, this study was the first to investigate the effect of *Auricularia polytricha* on macrophage polarization and anti-human breast cancer cells (MDA-MB-231 and MCF-7 cell lines). The human monocytic cell line (THP-1) was differentiated into macrophages by Phorbol-12-myristate-13-acetate (PMA) and polarized by various treatments. After the polarization state, cells were characterized by surface markers, gene expression, cytokine production, and protein expression. The outcomes of this study provide valuable information for AP and polarized macrophages on human breast cancer cells, which are crucial for a breast cancer drug and adjuvant development.

## Materials and methods

### Chemicals and reagents

*Auricularia polytricha* mushroom was purchased from Chang Daeng mushroom farm in Samutprakarn province, Thailand. Dulbecco’s Modified Eagle’s Medium (DMEM), Roswell Park Memorial Institute (RPMI)-1640 medium, phosphate buffered saline (PBS), trypsin 0.25% (v/v), and antibiotics were purchased from HyClone, Utah, USA. Fetal Bovine Serum (FBS), 3-(4,5-Dimethyl-2-thiazolyl)-2,5-diphenyl-2H-tetrazolium bromide (MTT), ergosterol, and propidium iodide were purchased from Sigma-Aldrich, Darmstadt, Germany. Hexane, ethanol, and dimethyl sulfoxide (DMSO) were purchased from RCI Labscan, Bangkok, Thailand. The flow cytometry reagents were purchased from BioLegend, California, USA. The human cytokine ELISA kits (IL-6, IL-1β, and TNF-α) were purchased from Thermo Scientific, Massachusetts, USA. Primary and secondary antibodies for Western blot analysis were purchased from Cell Signaling Technology, Massachusetts, USA. Bradford protein assay was purchased from Bio-Rad Laboratories, USA. Matrigel® and transwell® inserts were purchased from Corning, Arizona, USA.

### Mushroom extraction and crude polysaccharide isolation

**Figure 1:**
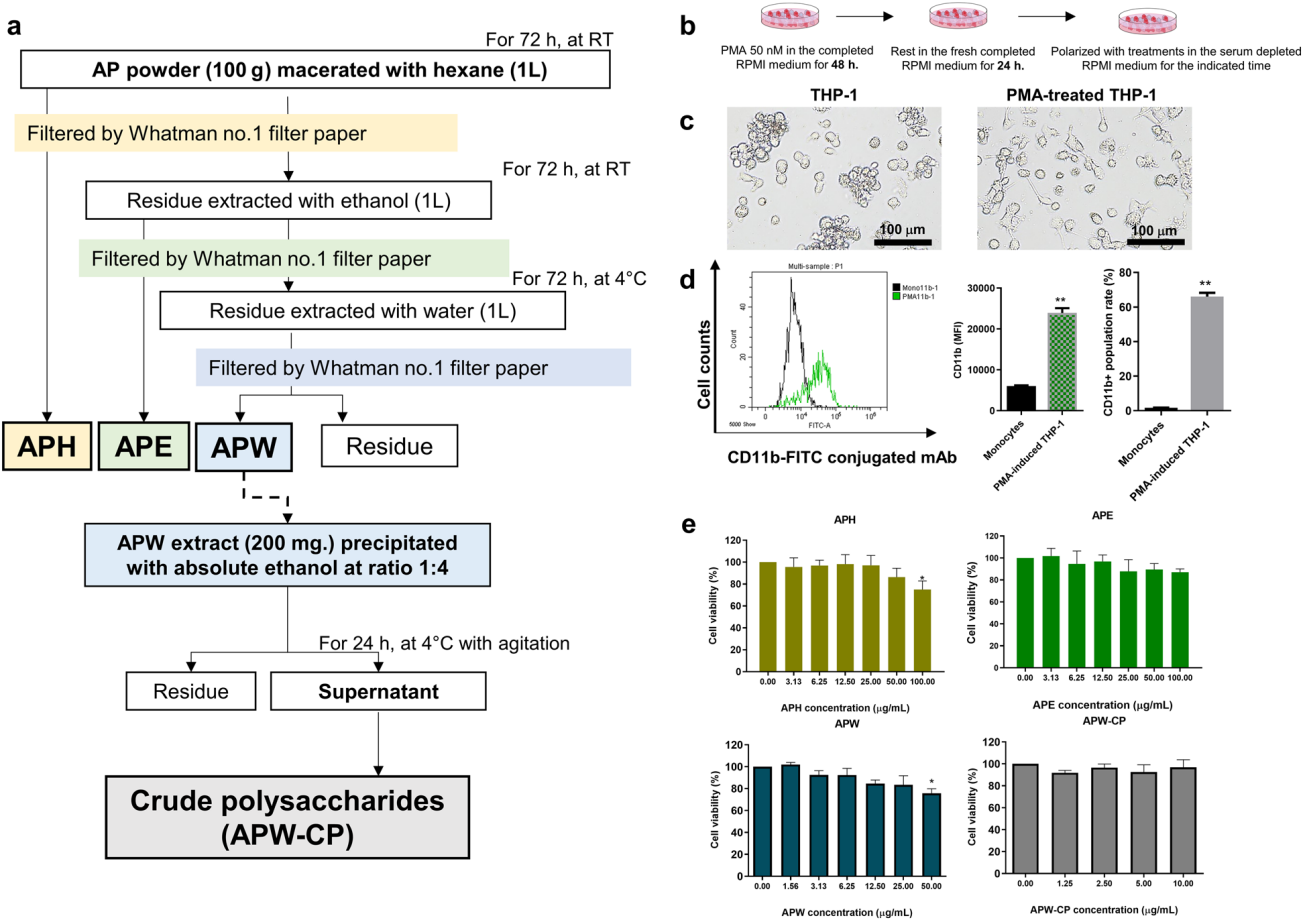
AP extraction, macrophage differentiation and cytotoxicity screening. (**a**) Mushroom extraction and crude polysaccharide isolation. (**b**) The polarization step (**c**) The morphological alteration of THP-1 derived macrophages after PMA activation captured by the phase-contrast microscope (scale bar; 100 μm). (**d**) Results from flow cytometry analysis showing the CD11b + surface marker expression after 48 h of PMA treatment (50 nM). (**e**) The cytotoxicity screening of THP-1-derived macrophages (M0) after 24 h of AP treatments; APH, APE, APW, and APW-CP. Data were expressed as mean ± SEM from triplicate experiments. The significant results were analyzed by Dunnett’s t-test, with * indicating *P* < 0.05 and ** indicating *P* < 0.001.

*Auricularia polytricha* extractions were performed using the sequential maceration described in our previous study^13^. AP powder was extracted with hexane at ratio of 1:10 (w/v) for 72 h. Then, ethanol was used to extract the residue for another 72 h at room temperature, followed by distilled water at 4 °C. The extract solution from hexane (APH) and ethanol (APE) was concentrated by the rotary evaporator at 100 rpm, 50 °C. On the contrary, the water extract (APW) was freeze-dried by the lyophilizer. Moreover, the crude polysaccharide of AP (APW-CP) was isolated from APW by the ethanol precipitation method (Fig. 1a). All extracts were kept at − 20 °C for further experiments. APH and APE were prepared in DMSO at a 100 mg/mL concentration, APW and APW-CP were stocked in sterile PBS at 50 mg/mL and 10 mg/mL, respectively. Stocks were aliquoted and stored at − 20 °C for further investigation. The vehicle control used in this study was DMSO (0.01%) for APH and APE extracts, whereas APW and APW-CP were PBS (0.01%).

### Measurement of mushroom glucans by enzymatic method

AP extracts, including APH, APE, APW, and APW-CP, were studied for their mushroom glucans content using β-Glucan (Yeast and Mushroom) Assay Kit (Megazyme International Limited, Bray, Ireland). The protocol was adapted from the manufacturer’s instruction by minimizing sample volume. The result of the glucans content was calculated using the Mega-Calc™ data calculator provided by the company.

### Fourier Transform Infrared Spectrometer (FT-IR)

The structural analysis of β-glucan was confirmed using FT-IR. The FT-IR analysis was subjected to Nicolet™ iS™5 FTIR Spectrometer coupled with iD7 ATR accessory (Thermo Scientific™, Wisconsin, USA). Samples were analyzed with 32 scans and resolution at 4 cm^−1^ between wavelength 4000–525 cm^−1^. Ambient air was subtracted from all samples as a background by OMNIC software (version 9.3.32, Thermo Scientific, Massachusetts, USA) (https://www.thermofisher.com/order/catalog/product/833-036200).

### Cell culture

The human monocytic cell line (THP-1, ATCC TIB-202) was maintained in RPMI-1640 supplemented with 10% fetal bovine serum (FBS) and 1% Penicillin–Streptomycin solution. The human breast cancer cells (MDA-MB-231, ATCC HTB-26 and MCF-7, ATCC HTB-22) were cultured in DMEM and supplemented with 10% FBS and 1% Penicillin–Streptomycin solution. All cell lines were generously gifted by Assistant Professor Dr. Viroj Boonyaratanakornkit (Department of Clinical Chemistry, Faculty of Allied Health Sciences, Chulalongkorn University). Cells were incubated in a humidified condition with 5% CO_2_ at 37 °C and used for experiments when reaching 80–90% confluency.

### Differentiation of THP-1 cells

THP-1 cells were cultured in RPMI-1640 supplemented with 10% FBS and 1% antibiotics until reaching 80–90% confluency. Cells were plated at 2 × 10^5^ cells per well in 12-well plates. To differentiate THP-1 monocytes to macrophages, PMA at 50 nM was added to the culture and incubated for 48 h. Morphological changes were observed under the phase-contrast microscopy. The non-adherent cells were washed out with PBS and the attached cells were maintained in PMA-free medium for another 24 h, as shown in Fig. 1b. Then, detection of CD11b surface marker was performed to distinguish between the macrophage (M0) and monocytes. After cells were scraped, they were washed with PBS, resuspended in a cell staining buffer and incubated with 5 μL of CD11b-FITC conjugated antibody for 30 min. Then, cells were stored on ice in the dark until the flow cytometric analysis.

### Macrophage polarization

Macrophages from differentiation step were polarized by inducing cells with multiple treatments in a 0.5% FBS medium. The distinctive genotypic and phenotypic characteristics of macrophage subpopulations were induced by LPS (10 pg/mL) and IFN-γ (20 ng/mL) for M1 macrophages and IL-4 (20 ng/mL) for M2 macrophages. In addition, the gene expression of polarized macrophages, cytokine production, and surface markers were examined.

### Cytotoxicity test

THP-1 cells (1 × 10^4^ cells per well) were plated into a 96-well plate followed by differentiation procedure. After the differentiation, various treatments were administered for 24 h. For cytotoxicity testing on human breast cancer cells (MCF-7 and MDA-MB-231), cells were plated at a density of 3 × 10^3^ cells per well before the 48 h of treatments. Then, the 20 μL of MTT (5 mg/mL) was added into each well, incubated for 3 h, and 150 ul of DMSO was added for formazan dissolving. Then, plates were read at 570 nm by Perkin Elmer 2300 EnSpire™ Multilabel Plate Reader (PerkinElmer, Massachusetts, USA). Non-toxic doses were selected to perform further experiments.

### Gene expression investigated by qRT-PCR

THP-1 were plated in a 12-well plate at 2 × 10^5^ cells per well, differentiated, and the polarization of macrophage was performed. After 12 h of polarization, cells were harvested, and RNA extraction was performed using GENEzol™ (Geneaid Biotech, New Taipei, Taiwan). RNA samples at a concentration of 1 µg were converted into cDNA by Maxime RT Premix Kit (iNtRON Biotechnology, Gyeonggi, Korea) and stored at − 20 °C until analysis. Quantitative RT-PCR (qRT-PCR) was used to measure the gene expression of human *CD86*, *CD206*, *IL6*, *TNFA*, *IL1B,* and *IL10*, while the *GAPDH* gene was used as the internal control. The qRT-PCR performances were monitored using RealMOD™ Green W 2 × qPCR Master mix (iNtRON Biotechnology) and cycling protocol with specific primers, as listed in Table 1S. The relative gene expression of interesting genes was calculated with the 2^−ΔΔ Ct^ formula.

### Cytokine production determined by ELISA

Pro-inflammatory cytokines released from polarized macrophages, including IL-6, TNF-α, and IL-1β, were measured by ELISA. Cells were polarized in 6-well cell culture plate and incubated for 24 h. The supernatants were collected into microtubes and centrifuged at 3000 × *g* for 10 min at 4 °C. Samples were stored at − 80 °C until analysis. The cytokine levels were measured following the manufacturer’s instructions (Thermo Fisher Scientific, Massachusetts, USA) and compared to assay kit standards.

### Surface marker determined by flow cytometry

The human CD11b, CD86, and CD206 surface markers were determined to assess the macrophage differentiation and polarization. Polarized cells were harvested and washed twice with a cold PBS and centrifuged at 1500 rpm for 5 min at 4 °C. Next, 10% FBS in PBS was used as blocking reagent and washing step was repeated. Then, resuspended cells in 100 μL of cell staining buffer; THP-1 cells (with and without PMA treatment) were stained with anti-human CD11b conjugated FITC (1:50). Additionally, M1 and M2 phenotypic markers were determined by CD86 and CD206 expression, respectively. Cells were stained with anti-human CD86 conjugated FITC and anti-human CD206 conjugated APC at dilution 1:50 and incubated on ice for 30 min in the dark. After the incubation period, cells were analyzed by the CytoFlex flow cytometer (Beckman Coulter, California, USA) for 5000 events per sample. The experiments were performed in triplicates.

### Protein expression determined by Western blot analysis

Western blot analysis was performed to investigate the NF-κB signaling pathway. Cells at 4 × 10^5^ cells per well were cultured in a 6-well plate. RIPA lysis buffer was added into the wells. Lysates were harvested after 1 h and centrifuged at 12,000 rpm for 20 min. Supernatants were collected into new tubes and stored at − 80 °C until analysis. Protein concentrations were performed and calculated using the Bradford protein assay (Bio-rad, California, USA). The denatured proteins (40 μg) were loaded into 10% SDS-PAGE. After transferring the protein to the Amersham™ Polyvinylidene fluoride (PVDF) membrane (Cytiva Amersham™, Buckinghamshire, UK), the membrane was blocked with 5% non-fat dried milk in TBST buffer for 1 h on a shaker and then incubated with antibodies. For protein expression detection, the membranes were incubated overnight with primary antibodies (1:1000) which are NF-κB p65 (#D14E12), p-NF-κB p65 (S536) (#93H1), IκB-α (#44D4) and p-IκB-α (S32) (#14D4). Then, membranes were probed with anti-rabbit IgG HRP-linked antibody (1:5000) for 1 h. GAPDH was determined and used as an internal control. Amersham™ ImageQuant™ 800 biomolecular imager (GE Healthcare, New Jersey, USA) was used to detect the signal through Amersham™ ECL Select™ reagent (Cytiva Amersham™, Buckinghamshire, UK). The band intensity was quantified by ImageJ software version 1.53a (http://imagej.nih.gov/ij).

### Conditioned medium collection

THP-1 cells (4 × 10^5^ cells per well) were seeded in a 6-well plate and the polarization steps were performed using various treatments. Supernatants as a conditioned medium (CM) were collected from these cultures by centrifugation at 3000 × g for 10 min at 4 °C and stored at − 80 °C until used. For culturing the cells, the ratio of CM and fresh completed medium was 1:1. To investigate the anti-tumor activity, cell viability, apoptosis, and cell cycle analysis were monitored.

### Apoptosis and cell cycle assays

Breast cancer cells (MDA-MB-231 and MCF-7) at 5 × 10^4^ cells were cultured with CM in 12-well plate. For the apoptosis assay, harvested cells were resuspended in 100 μL of binding buffer. Then, Annexin V-FITC and PI solutions were added and incubated on ice for 30 min in the dark. Next, samples were transferred to the flow tubes, 400 μL of the binding buffer was added. For the cell cycle analysis, harvested cells were washed with PBS, 70% ethanol was added while vortexing to fix the cells. After 30 min incubation on ice, cell pellets were washed twice with PBS. Finally, the 500 μL of PI solution (0.1%, v/v Triton X-100; 10 µg/mL propidium iodide; 100 µg/mL DNase-free RNase A in PBS) were added and cells were incubated for 30 min in the dark on ice until analysis. The CytoFLEX™ flow cytometer (Beckman Coulter, California, USA) was used to analyze the apoptotic cells and cell phases by collecting at least 5000 cells per sample.

### Transwell invasion assay

To investigate the effect of polarized macrophages on breast cancer cell invasion, the transwell invasion experiments were performed. The lower compartment of the 24-well plate was seeded with THP-1 cells followed by the polarization steps. After the polarization was completed, the upper inserts (pre-coated with 50 µL of diluted Matrigel at a 1:8 ratio in serum-free RPMI and solidified in the incubator for 1 h) were seeded with human breast cancer cells (5 × 10^4^ cells per well). The assay was determined at 24 h. Non-invaded cells were removed from the inserts by cotton swaps and fixed with 100% methanol for 20 min. Then, cells were washed with PBS and stained with 0.4% crystal violet for 20 min. After re-washing with PBS and air drying, chambers were photographed under the inverted microscope (ZEISS, Oberkochen, Germany) at 100 × magnification for five random fields per insert.

### Statistical analysis

All experiments were performed in triplicates. Data were shown as the mean ± standard error of the mean (SEM). One-way analysis of variance (ANOVA) was used to analyze the significant results using SPSS software version 22.0 (https://www.ibm.com/spss). A post hoc Dunnett’s test with *P value < 0.05 and **P value < 0.001 were considered significant results.

## Results

### Differentiation of THP-1 monocytic cells into macrophage-like cells

To investigate the macrophage polarization, the human monocytic THP-1 cell line was used as the macrophage model. After PMA activation for 48 h, the morphological changes of THP-1 cells could be detected with cell attachment and elongation (Fig. 1c). CD11b, a macrophage marker, was also examined to confirm macrophage differentiation. Results showed that PMA could elevate the expression of CD11b + FITC population from 1.67 ± 0.11% to 66.01 ± 2.25% of the cell population compared to the non-PMA treated THP-1 cells. Moreover, the mean fluorescence intensity (MFI) of CD11b + overlayed histogram showed that the PMA-treated cells were significantly expressed compared to the non-PMA treated group (Fig. 1d). Therefore, THP-1 derived macrophages exhibited the macrophage-like phenotype (M0) with CD11b surface marker expression and were favorable for polarization experiments.

### Cytotoxicity of AP on THP-1 derived macrophages (M0)

To evaluate the effect of AP extracts on THP-1derived macrophages, the cytotoxicity screening of extracts after macrophage differentiation to M0 was performed. THP-1 derived macrophages were treated with APH, APE, APW, and APW-CP extracts for 24 h. MTT was added to measure the cell cytotoxicity. Results showed that APH and APE (3.13–50 μg/mL) as well as APW-CP (1.25–10 μg/mL) were not toxic to the cells, whereas APW at 50 μg/mL showed significant cytotoxicity (Fig. 1e). Thus, the extracts at specific concentration; APH, APE, APW (1.25–5 μg/mL) and APW-CP (1.25 and 2.5 μg/mL) were selected for further experiments.

### APW and APW-CP extracts induced M1-related gene expression and pro-inflammatory cytokine production

**Figure 2:**
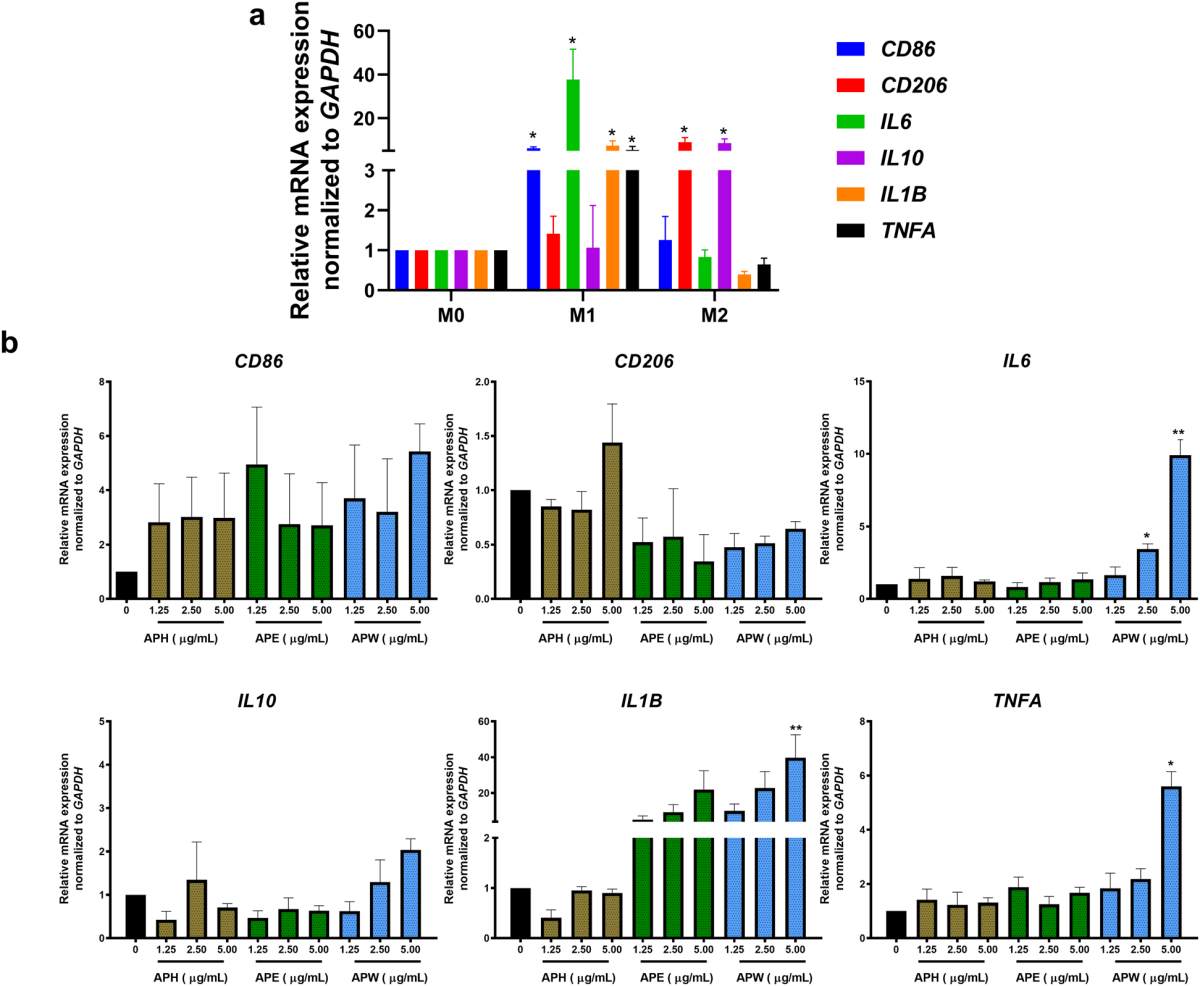
The pro-inflammatory cytokine gene expression (*CD86*, *CD206*, *IL6*, *IL10*, *IL1B,* and *TNFA*) by macrophages after polarization for 12 h. (**a**) The treatment of DMSO for M0, LPS (10 pg/mL) + IFN-γ (20 ng/mL) for M1, and IL-4 (20 ng/mL) for M2, respectively. (**b**) The treatment of APH, APE, and APW, respectively. Results were collected in three independent experiments and expressed as mean ± SEM. The significant results were analyzed by Dunnett’s t-test, with * indicating *P* < 0.05 and ** indicating *P* < 0.001.

The resting-state macrophages (M0) were induced by either M1 or M2 inducers as positive controls; LPS (10 pg/mL) + IFN-γ (20 ng/mL) for M1 and IL-4 (20 ng/mL) for M2. Cells were induced and gene expressions were checked at various time points including 6, 12 and 18 h. After the incubation period, M1-related genes (*CD86*, *IL6*, *IL1B*, and *TNFA*) and M2-related genes (*CD206* and *IL10*) were determined by qRT-PCR. The result showed that M0, M1, and M2 populations displayed different gene patterns, as shown in supplementary Fig. 1S. M1 inducer could regulate the expression of *IL6* and *IL1B* after 6 h and CD86 after 12 h, significantly compared to M0. Meanwhile, the M2 inducer also significantly upregulated *CD206* and *IL10* genes, after 12 h of polarization. From these results, a 12 h was selected as an optimal incubation period for AP effect on macrophage polarization, as shown in Fig. 2a.

**Figure 3:**
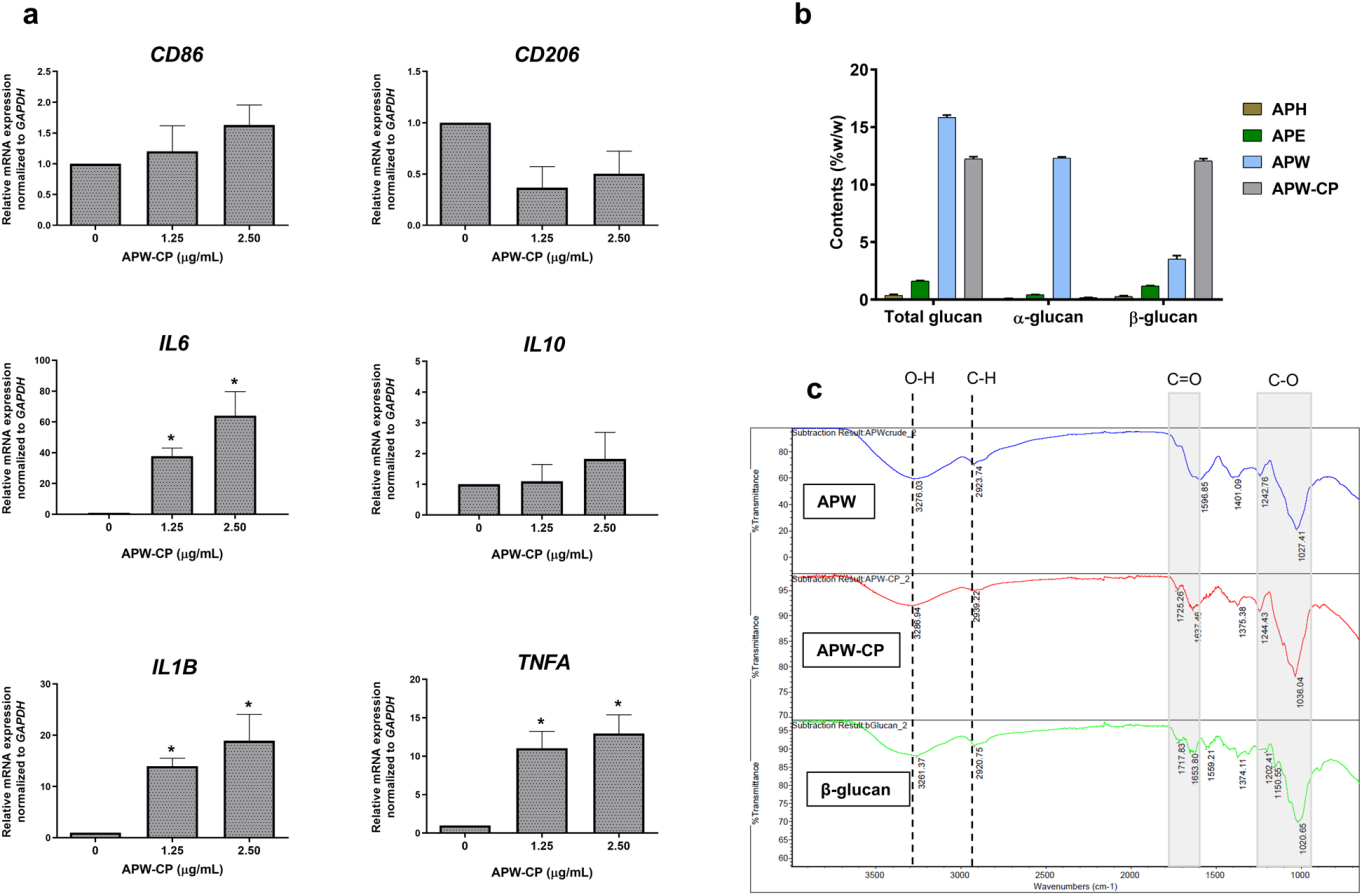
The polarization effect of APW-CP and β-glucan determination. (**a**) The gene expression of macrophages after being treated with APW-CP for 12 h. (**b**) The analysis of β-glucan contents in AP extracts by enzymatic commercial kit. (**c**) The FT-IR spectrum of APW, APW-CP, and β-glucan yeast control. Results were collected in three independent experiments and expressed as mean ± SEM. The significant results were analyzed by Dunnett’s t-test, with * indicating *P* < 0.05 and ** indicating *P* < 0.001.

The effect of AP extracts (APH, APE, and APW at 1.25–5 μg/mL) on macrophage polarization was studied. We found that APW could modulate *IL6*, *IL1B,* and *TNFA* gene expression significantly in a dose-dependent manner. Also, APW-CP (1.25 and 2.5 μg/mL) significantly upregulated expression of *IL6*, *IL1B,* and *TNFA* genes in a dose-dependent manner (Fig. 3a). APW and APW-CP induced M0 to exhibit M1-like phenotypes and suppressed M2-like phenotypes. These results suggest that APW and APW-CP may contain immunomodulatory substances inducing M1 macrophages.

### Potential role of β-glucan in APW and APW-CP for M1 polarization

In this study, we hypothesized that β-glucan in AP extracts may be the lead compound for M1 polarization. Hence, the enzymatic assay of mushroom glucans was performed to investigate the β-glucan content in all AP extracts. APW and APW-CP composed of higher β-glucan content compared to APH and APE, as shown in Table 1 and Fig. 3b. Interestingly, APW-CP obtained by ethanol precipitation from APW contained β-glucan content three times higher than AP. Existed β-glucan in APW-CP supports the polarization effect on macrophages.

**Table 1:** The mushroom β-glucan content was measured by enzymatic method.

Samples	mg/g extract dry weight (± SEM)		
	Total glucans	α-glucan	β-glucan
APH	3.75 ± 0.66	1.08 ± 0.03	3.51 ± 0.66
APE	16.2 ± 0.40	4.3 ± 0.11	11.9 ± 0.32
APW	158.42 ± 1.21	123.11 ± 0.58	35.31 ± 1.78
APW-CP	119.18 ± 1.40	1.82 ± 0.08	117.36 ± 1.32

All samples were collected and measured using β-Glucan (Yeast and Mushroom) assay kit in triplicates. The calculated results were expressed as mean ± SEM.

### Characterization of β-glucan in AP extracts by FT-IR spectral analysis

FT-IR technique was performed to prove the presence of β-glucan in APW and APW-CP. β-glucan yeast control was subjected to FT-IR as the standard and spectrums at the wavelength range of 4000–525 cm^−^1 were compared to APW and APW-CP (Fig. 3c). The board region at 3200–3400 cm^−1^ was found in all three specimens assigned as the O–H stretching. The polysaccharides, the long-chain polymeric carbohydrates, exhibited vibration characteristics in the range of 1600–1700 cm^−1^ with C=O stretching. In addition, Band intensity at 1000–1200 cm^−1^ strongly indicated the presence of the β-glycosidic C–O bond^14^. The yeast 1,3:1,6 -β-D-glucan showed the specific band at 890 cm^-1^ at the anomeric region^15^. APW-CP with the highest β-glucan content, displayed a noticeable 890 cm^−1^ peak compared to APW. Herein, IR spectra confirmed the β-D-glucan in APW and APW-CP as the main active component in this study.

### Characterization of APW and APW-CP polarized macrophages by surface marker determination and cytokine production

**Figure 4:**
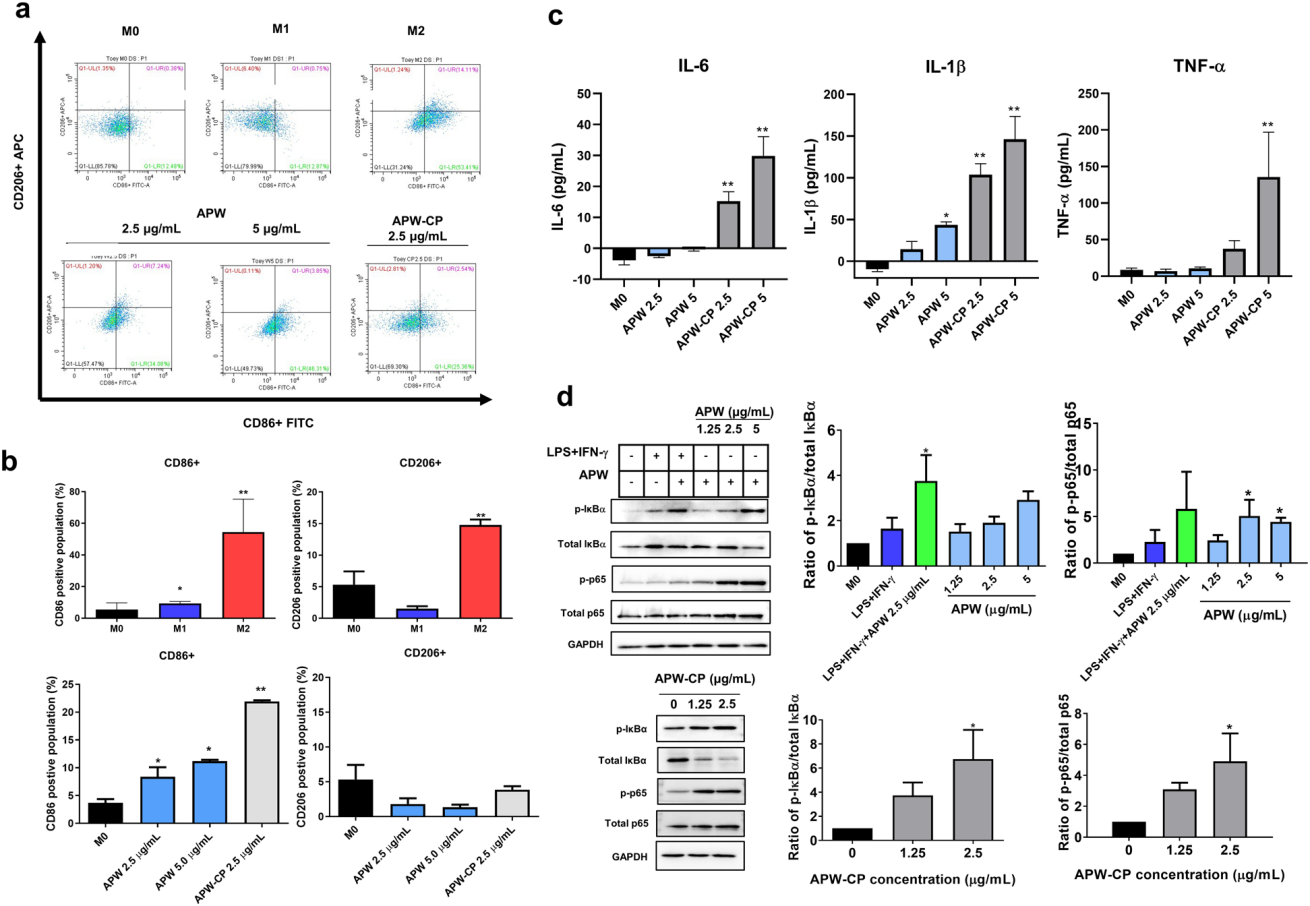
The characteristics of polarized macrophages after 24 h. (**a**) The representative dot plots of surface markers (CD86 and CD206) and (**b**) the positive cell population by flow cytometry. (**c**) The IL-6, IL-1β, and TNF-α levels of conditioned medium from polarized macrophages. (**d**) NF-kB signaling markers (p-p65, total p65, p-IκB, total IκB, and GAPDH) were detected by Western blotting after 1 h of polarization. Results were collected from three independent experiments and expressed as mean ± SEM. The significant results were analyzed by Dunnett’s t-test, with * indicating *P* < 0.05 and ** indicating *P* < 0.001. The original blots are presented in Supplementary Fig. 4.

To characterize the polarization states of macrophages phenotypic confirmation by surface markers was required. After the polarization, we found that the M1 phenotype exhibits high CD86 and low CD206. In contrast, M2 displayed co-expression of CD206 and CD86. Additionally, APW at 2.5 μg/mL could significantly increase CD86 expression of M0 from 3.68 ± 0.68 to 8.38 ± 1.71% and suppressed CD206 expression (Fig. 4a,b). On the other hand, APW-CP (2.5 μg/mL) induced higher expression of CD86 (21.92 ± 0.22%). These results indicated that APW and APW-CP could polarize M0 to M1 phenotype. We also confirmed the characteristic of M1 by measuring the IL-6, IL-1β, and TNF-α cytokine production. APW-CP (2.5 and 5 μg/mL) significantly stimulated IL-6, and IL-1β cytokine production but TNF-α was significantly elevated at 5 μg/mL of APW-CP. Furthermore, APW at 2.5 and 5 μg/mL induced a trace amount of cytokine production. However, APW at 5 μg/mL significantly induced IL-1β level (Fig. 4c). Our results imply that β-glucan in APW-CP played a role in M1 polarization on THP-1-derived macrophages.

### APW and APW-CP induced M1 polarization via NF-κB signaling pathway

The previous study of macrophage polarization demonstrated that M1 activation could accelerate the pro-inflammatory cytokine production via NF-κB p65/p50 activation and suppress the transcription factor of the STAT6, leading to M2 suppression^16^. To determine the NF-κB signaling pathway influenced by AP extracts. Polarized cells were collected and proteins in both unphosphorylated and phosphorylated forms were analyzed after 1 h (Fig. 4d). Western blot results suggested that APW could induce the phosphorylation of IκB-α and significantly cascade the p65 phosphorylation in a dose-dependent manner. On top of that, the co-treatment between M1 (LPS + IFN-γ) and APW (2.5 μg/mL) exhibited an additive effect on IκB-α phosphorylation significantly. APW-CP at 2.5 μg/mL triggered a robust NF-κB p65 phosphorylation up to six times higher than the M0 state. Therefore, APW and APW-CP stimulated NF-κB signaling, resulted in cytokine genes and M1 polarization.

### Induction of cytotoxicity and apoptosis in breast cancer cells by CM from AP-polarized macrophages

**Figure 5:**
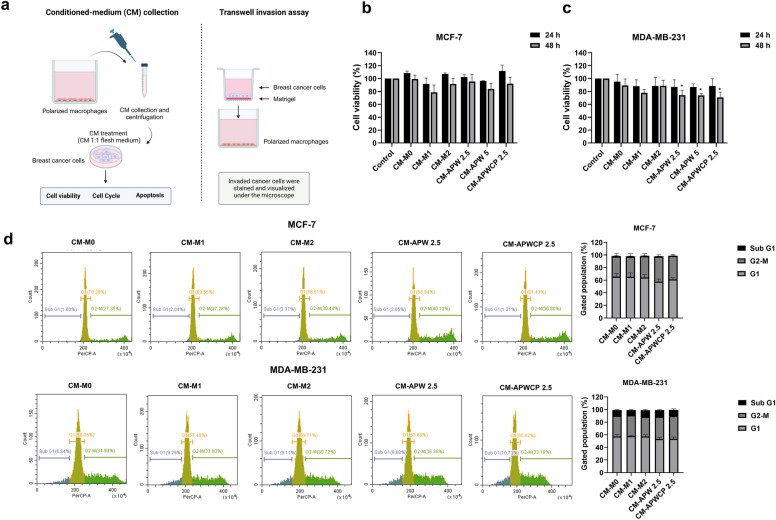
Anti-tumor activity of polarized macrophages. (**a**) The schematic assessment of polarized macrophages for anti-tumor activity in the breast tumor microenvironment by conditioned medium collection and transwell invasion assay. (**b**) The cytotoxicity screening of polarized macrophages-derived conditioned medium on MCF-7 and (**c**) MDA-MB-231 by MTT assay. (**d**) The cell cycle analysis of polarized macrophage-derived conditioned medium on breast cancer cells for 48 h by flow cytometry. The cell cycle population was carried out by PI staining and gated as sub-G1, G1, and G2-M populations. Results were collected in three independent experiments and expressed as mean ± SEM. The significant results were compared with the control cells for MTT assay and CM-M0 for cell cycle assay, then analyzed by Dunnett’s t-test with * *P* < 0.05 and ** *P* < 0.001.

**Figure 6:**
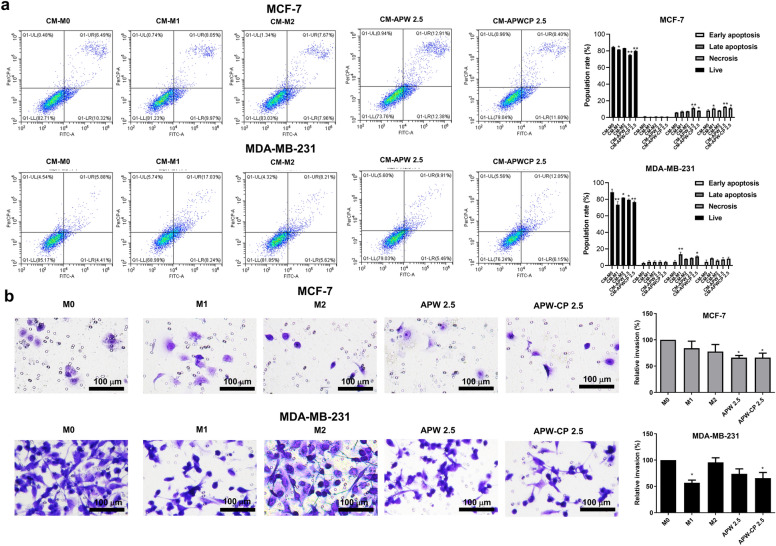
The apoptosis and invasion of breast cancer cells against polarized macrophages. (**a**) Representative dot-plots of annexin V-PI double staining for apoptosis assay and cell population gated as live, early, late, and necrosis. (**b**) The transwell invasion assay of MCF-7 and MDA-MB-231 after conditioned medium treatments for 24 h with 100× magnifications (scale bar; 100 μm). Results were collected in three independent experiments and expressed as mean ± SEM. The significant results were compared with CM-M0 and then analyzed by Dunnett’s t-test with * *P* < 0.05 and ** *P* < 0.001.

After 24 h of polarization state, conditioned medium from polarized macrophages were collected and incubated with breast cancer cells (Fig. 5a). Cell viability, apoptosis, and cell cycle assays were determined after conditioned medium (CM) treatment. The cytotoxicity screening of MDA-MB-231 and MCF-7 cells was detected after 24 and 48 h after CM exposure. Results showed that the CM from APW-polarized macrophages (CM-APW) (2.5 and 5 μg/mL) and APW-CP (CM-APWCP) (5 μg/mL) significantly inhibited MDA-MB-231 cell viability at 48 h. Furthermore, cell cycle and apoptosis assays were performed after 48 h of CM treatments. The CM was slightly arrested the cells at G2-M subpopulations in both cell lines. Interestingly, apoptosis of breast cancer cells was significantly induced in APW and APW-CP conditioned-medium treatments (Fig. 6a). This suggests that conditioned medium derived from AP-polarized macrophages showed the anti-tumor effect towards breast cancer cell lines via apoptosis induction and cytotoxicity.

### Suppression of breast cancer cell invasion by AP-polarized macrophages using the transwell co-culture experiment

The effect of polarized macrophages was further investigated via a transwell invasion assay to mimic the breast cancer cell microenvironment. After 24 h of co-culturing of polarized macrophages and breast cancer cells, invaded cells were stained and photographed. The relative invasion rate was calculated compared to control cells (M0). Results showed that the invasiveness of MDA-MB-231 was significantly inhibited by M1 and APW-CP (2.5 μg/mL) polarized macrophages. Additionally, APW and APW-CP significantly inhibited MCF-7 invasion compared to M0 (Fig. 6b). However, the inhibition of breast cancer cell invasion might come from the direct effect of APW-CP itself. To reveal the effect of APW-CP on breast cancer cell invasion, the cytotoxicity assay of APW and APW-CP were performed on breast cancer cells. Results showed that APW (20 μg/mL) and APW-CP (5–20 μg/mL) could  significantly inhibit MCF-7 cell viability, while MDA-MB-231 cell viability was not affected by APW and APW-CP exposure up to 20 μg/mL, as shown in supplementary Fig. 2a–d. Moreover, APW and APW-CP treatments at 2.5 μg/mL showed no direct effect on MDA-MB-231 (the highly invasive breast cell line) invasion. These findings indicated that the inhibitory effect on breast cancer cells was influenced by polarized macrophages (supplementary Fig. 2e,f). Therefore, the invasion assay demonstrated the anti-tumor effect of inducible M1-polarized macrophages in the breast cancer cell microenvironment, further strengthening AP’s role as an immunomodulator in tumor immunotherapy.

## Discussion

The TME is composed of stromal fibroblasts, endothelial cells, immune cells, and non-cellular components that harbor tumorigenesis and progression^17^. Immune cells are crucial for disease progression because of the intracellular networks interacting with cytokines, chemokines, growth factors, and signal mediators secreted by immune cells^18^. The infiltration of immune cells around the tumor site could advance the prognosis depending on tumor types and stages^19^. Among immune cells, TAMs dominate TME and contribute to immunosuppression. Macrophages are generally categorized in an innate immune system with neutrophils, dendritic cells, and monocytes which play a significant role in host defense against foreign organisms^20^. In contrast, the adaptive immune system consists of T and B lymphocytes that directly eliminate the tumor by antigen-presenting cells^21^. Macrophages contain two main phenotypes, including M1 (a classical activated macrophage), and M2 (an alternative activated macrophage). M1 macrophages commonly exhibited the pro-inflammatory state with T-helper cell type 1 (Th1) activation by secretion of ROS, IL-6, IL-1β, and TNF-α. When infiltrated with M1 macrophages, tumors showed a good prognosis and were susceptible to cancer treatment^22^. On the other hand, the M2 phenotype was often raised as the TAMs mimicked phenotype since it activated immunosuppressors and tumor growth factors such as TGF-β, IL-10, PD-L1, VEGF, and MMPs^23,24^. TAMs have been reported to increase resistance to treatment in many cancer types, including lung, breast, and colorectal cancers^25–27^. Targeting TAMs in tumor sites showed anti-tumor effects through modulation of immunity, including TAMs blocking, depletion, and repolarization to M1^28^. This study utilized the human monocytic cell line (THP-1) as a model of macrophage polarization. Macrophages derived from THP-1 cells could be generated from PMA activation, morphological changes and macrophage marker (CD11b), a surface marker, for macrophage differentiation were observed^29^. In our study, THP-1 with PMA activation for 48 h showed the adherence of cells and the significant expression of CD11b marker on the cell surface (Fig. 1c,d). These results indicate that the macrophage model (M0) was well established. Furthermore, all four extracts (APH, APE, APW, and APW-CP) were screened for cytotoxicity against the M0 stage. We found that AP extracts at 10 μg/mL showed no effect on macrophage survival and was selected for further experiments. These findings were consistent with the cytotoxicity screening of AP extracts on murine macrophage RAW 264.7 cells which exhibited cellular toxicity at 500 μg/mL^30^.

LPS and IFN-γ have been reported to prime M1 phenotypes through TLR4/NF-κB signaling pathway and regulate the pro-inflammatory cytokines such as IL-6, TNF-α, and IL-1β and surface makers like CD80 and CD86^31^. IL-4 also induced the expression of CD206 surface marker, the mannose receptor C type 1, and IL-10 secretion^32^. The upregulation of *CD86*, *IL6*, *IL1B,* and *TNFA* genes was detected after 12 h of LPS + IFN-γ activation. *CD206* and *IL10* genes for M2 phenotype were exhibited with IL-4 exposure (Fig. 2a). These results demonstrate that M1 and M2 could be distinguished by their specific patterns. To determine the effect of AP extracts and to find the effective fractions for macrophage polarization, a 12 h incubation period was selected. Results showed that APW and APW-CP significantly elevated the M1-related cytokine gene expression in a dose-dependent manner. Moreover, the effects of APW and APW-CP were related to the β-glucan content (Fig. 3b).

β-glucan was identified as the active compound in APW and APW-CP, as confirmed by the enzymatic method and FT-IR analysis. From FT-IR data, the absorption peaks near 1160, 1078, 1048, and 890 cm^−1^ indicated the β-configuration of polysaccharides or mushroom 1,3:1,6 -β-D-glucan^33^. We found the correlative pattern between APW-CP, the suspected β-D-glucan enrichment fraction, and the standard β-D-glucan. Moreover, the distinct 890 cm^−1^ peak in APW-CP could be visualized more easily than in APW due to the substance purity.

Macrophages show distinguished expressions of phenotypes on the cell surface and produce specific cytokines depending on the activators. The surface markers (CD86 and CD206) and cytokine levels (IL-6, IL-1β, and TNF-α) were determined to investigate the macrophage polarization, as shown in Fig. 4a–c. In our study, M1, APW, and APW-CP showed the significant expression of CD86 but the suppression of CD206. On the contrary, M2-like phenotypes polarized by IL-4 exhibited the co-expression of CD86 and CD206. However, this outcome has also been found in a previous study on a malarial infection, showing that IL-4 could regulate the surface markers of CD86 and CD206 but not alter the phagocytic activity and Th1 cytokines specialized for M1 phenotype^34,35^. As previously mentioned, β-glucan was proposed as the bioactive compound in AP to boost M1 polarization. In agreement with previous works, researchers revealed that polysaccharides and β-glucan from *Saccaromyces cerevisiae* portrayed the immunomodulatory effects on macrophages, governed the adaptive immune system and tumor suppression^36–38^.

The underlying mechanism of M1 polarization was involved in the NF-κB signaling pathway, TNF signaling pathway, and viral myocarditis^39^. NF-κB is the critical regulator of inflammation and immunity. Immune cells that express the pathogen-associated molecular patterns (PAMPs), including toll-like receptors (TLRs), RIG-I-like receptors, NOD-like receptors (NLRs), and C-type lectin-like receptors, could recognize different damage-associated molecular patterns (DAMPs) and cascade the molecular signaling^40^. NF-κB signaling contains two significant pathways (canonical and non-canonical). The canonical pathway is mainly modulated by IκB-α degradation. Then, the IκB kinase (IKK) complex releases the NF-κB Rel A/p50 dimers into the nucleus and transcripts the responsible genes, such as *IL1B*, *IL6,* and *TNFA*^41^. In contrast, a non-canonical pathway requires the process of p100 instead of IκB-α and translocates the active Rel B/p52 complex to the nucleus^42^. Correspondingly, M1 macrophages processed the pro-inflammatory responses via NF-κB to mediate other immune cells. M1 signaling influences T-cell activation and differentiation of Th1 and Th17 cells^43^. In this study, APW and APW-CP regulated the Th1 cytokine genes through the NF-κB canonical pathway (NF-κB p65/ IκB-α), as shown in Fig. 4d. Interestingly, the effect of APW and APW-CP on M1 polarization was explained by previous evidence from β-glucan study. β-glucan could bind to the dectin-1 receptor on macrophage and induce the downstream targets of Th1. However, dectin-1 triggers an inflammatory response co-stimulated by the extracellular TLR2/4 activation^44,45^. Our findings suggest that the modulation of APW and APW-CP on NF-κB signaling prone cells to M1 phenotypes.

In this study, the effect of M1-trained macrophages on human breast cancer cells: estrogen receptor-positive cell line (MCF-7) and triple-negative cell line (MDA-MB-231) were investigated. Breast cancer progresses in patients with M2 domination, but M1 polarization portrayed a better outcome^46,47^. Presently, we found that the conditioned medium collected from APW and APW-CP-trained macrophages (CM-APW and CM-APWCP) significantly inhibited MDA-MB-231 cell survival after 48 h of incubation. This result correlated with the apoptosis population of breast cancer cells in Fig. 6a. Accordingly, the transwell invasion assay was also performed to determine breast cancer cell invasion. Results manifested that M2-like macrophages enhanced the aggressiveness of breast cancer cells, especially in MDA-MB-231. M1 and APW-CP polarized conditions significantly repressed the MDA-MB-231 invasion, as displayed in Fig. 6b. In addition, polysaccharides, active compounds in the present study, displayed the anti-tumor activity in previous studies. For example, crude polysaccharides isolated from soybean induced the cell cycle arrest at the G0/G1 phase on liver and cervix cancer cells at the dose of 50 μg/mL^48^. Besides, polysaccharides from ginger (400 μg/mL), *Artemisia sphaerocephala* (50–200 μg/mL) and slug (500 μg/mL) showed anti-proliferation and induced apoptosis on liver ^49^, gastric^50^, lung and breast cancer cells^51^, respectively. Our findings on the direct effects of crude polysaccharides against MCF-7 and MDA-MB-231 demonstrated that APW (20 μg/mL) and APW-CP (5 μg/mL) significantly inhibited MCF-7 but not MDA-MB-231 viability (Supplementary Fig. 2a,b). APW (1.25–5 μg/mL) and APW-CP (1.25 and 2.5 μg/mL) showed no direct effect on both MCF-7 and MDA-MB-231 cells but conditioned medium from AP-polarized macrophages exerted anti-tumor effects on MCF-7 and MDA-MB-231. The immunomodulation and cytotoxic effect from APW and APW-CP possibly improve the efficacy of breast cancer treatment.

In TME niches, especially in breast cancer, each breast cancer subtype responds to the environment differently. Matrix metalloproteinase-9 (MMP-9) is involved in cell remodeling in the extracellular matrix, which plays a crucial role in cancer invasion and metastasis^52^. In breast cancer, MMP-9 acts as the prognostic marker for breast cancer progression^53^. The capability of breast cancer invasion was reduced because of the MMP-9 level in the microenvironment. M2 phenotypes expressed and secreted MMP-9 enzymes to the extracellular matrix and supported the invasion of breast cancer^27^. Our present work has determined the gene expression of *MMP9* on MDA-MB-231 after treated with a conditioned medium derived from M0, M1 and M2 macrophages (CM-M0, CM-M1 and CM-M2). We found that CM-M2 treatment after 24 h could upregulate *MMP9* relative expression of MDA-MB-231 cells significantly when compared to CM-M0 and CM-M1, as shown in supplementary Fig. 2g. Additionally, APW-CP showed promising results in M1 polarization and inhibited MDA-MB-231 cell invasion. The MMP-9 and TGF-β are M2 macrophage markers and play roles in tumor progression^54^. To clarify the effect of APW-CP treatment on polarized macrophages, the gene expression of *MMP9* and *TGFB1* was performed by qRT-PCR. As shown in Supplementary Fig. 3a, we found that *MMP9* and *TGFB1* were upregulated in M2 macrophages significantly. Meanwhile, APW and APW-CP-trained macrophages retained *MMP9* and *TGFB1* gene expression on THP-1 cells (Supplementary Fig. 3b,c). These evidences supported data from invasion assay that M1 and APW-CP polarized macrophages inhibited breast cancer cell invasion. Hence, APW-CP treatment could modulate macrophage to M1 and suppress MMP-9 production.

Recently, there has been an impetus in cancer immunotherapy due to the recurrence of cancer and treatment resistance in patients^55^. Furthermore, adjuvant treatment is expected to help the patient’s immune system fight the remaining cancerous cells and maintain the host defense. Based on our study, M1-trained macrophages by APW-CP could reduce the invasiveness of breast cancer cells. However, further investigation on in vivo model should be continued. The application of AP, the source of β-glucan, could be used as an adjuvant therapy for breast cancer.

### Supplementary Information

Supplementary Figures.

Supplementary Tables.

## Data Availability

Data is provided within the manuscript or supplementary information files.
