# Supplementary material for: M1 macrophages polarized by crude polysaccharides isolated from Auricularia polytricha exhibit anti-tumor effect on human breast cancer cells
Source: Sci Rep. 2024 Apr 8;14:8179. doi: 10.1038/s41598-024-58208-2 (PMC11001921; doi:10.1038/s41598-024-58208-2)
Supplement: Supplementary file 1 — Supplementary Figures. [file 41598_2024_58208_MOESM1_ESM.pdf]

Supplementary Figures

Title

M1 macrophages polarized by crude polysaccharides isolated from *Auricularia polytricha* exhibit anti-tumor effect on human breast cancer cells

Authors

Sunita Nilkhet[1], Kuljira Mongkolpobsin[2], Chanin Sillapachaiyaporn[3], Nichaporn Wongsirojkul[2], Tewin Tencomnao[3] and Siriporn Chuchawankul[2,4 *]

Affiliations

[1] Program in Clinical Biochemistry and Molecular Medicine, Department of Clinical Chemistry, Faculty of Allied Health Sciences, Chulalongkorn University, Bangkok, 10330, Thailand.

[2] Department of Transfusion Medicine and Clinical Microbiology, Faculty of Allied Health Sciences, Chulalongkorn University, Bangkok 10330, Thailand.

[3] Department of Clinical Chemistry, Faculty of Allied Health Sciences, Chulalongkorn University, Bangkok, 10330, Thailand.

[4] Immunomodulation of Natural Products Research Unit, Chulalongkorn University, Bangkok, 10330, Thailand.

[*] Corresponding author: Siriporn.ch@chula.ac.th (S.C.)

Supplementary Figure 1

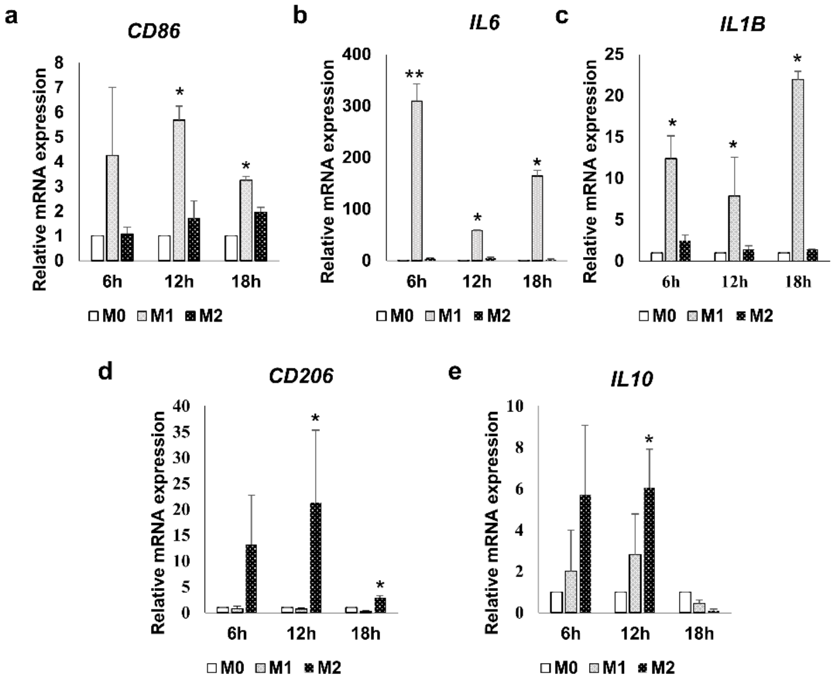

Supplementary Figure 1 Time optimization for M0, M1 and M2 macrophage polarization related genes analyzed by qRT-PCR. The relative gene expression of M0, M1 and M2 macrophages after 6, 12 and 18 h of incubation. (a) *CD86* (b) *CD206* (c) *IL1B*, (d) *CD206*, (e) *IL10* genes were performed and normalized to *GAPDH* and expressed as mean ± SEM. The significance result was analyzed by Dunnett's t-test with * $P < 0.05$ and ** $P < 0.001$.

.

Supplementary Figure 2

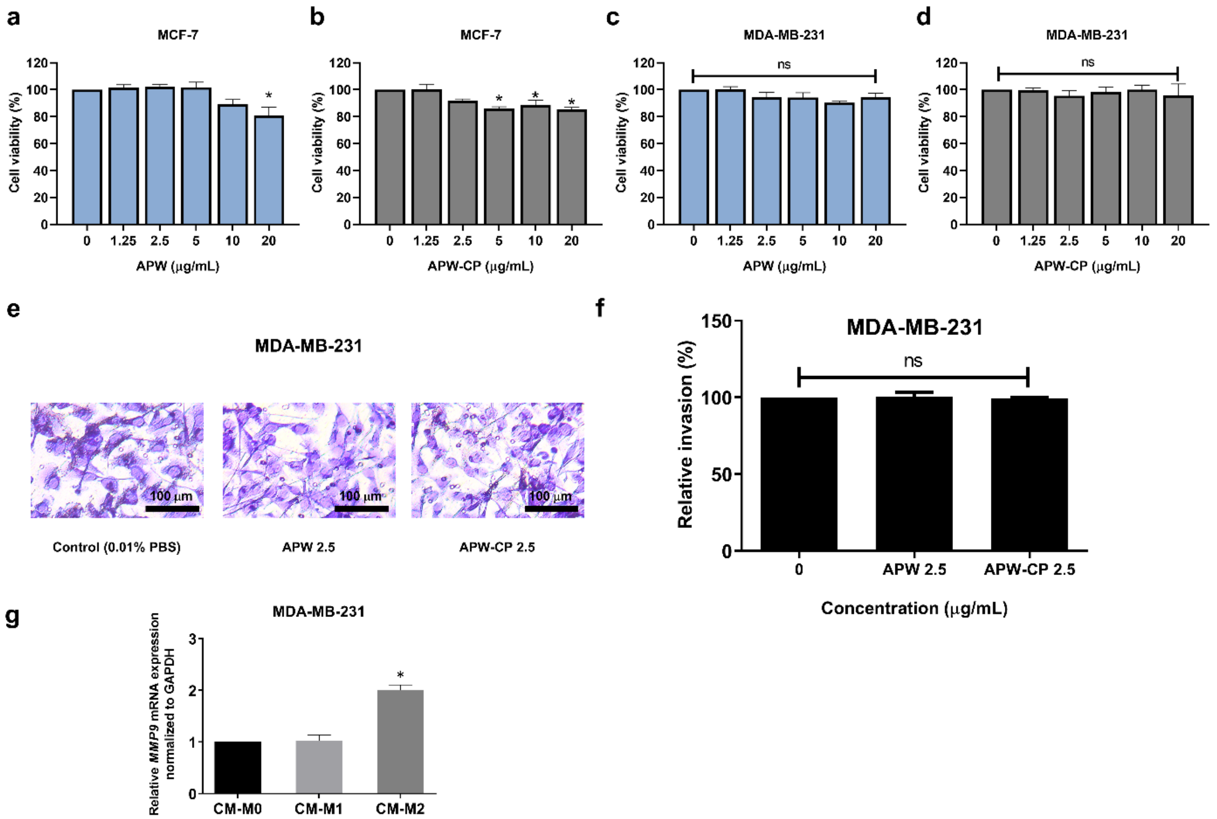

Supplementary Figure 2 The direct effect of AP extracts on human breast cancer cells. The cytotoxicity screening of APW and APW-CP extracts on (a and b) MCF-7 and (c and d) MDA-MB-231 cells after 48 h by MTT assay. (e) The transwell invasion assay of MDA-MB-231 cell with APW (2.5 μg/mL) and APW-CP (2.5 μg/mL) treatments after 24 h. Then, invaded cells were stained by crystal violet and photographed for five fields per insert (scale bar; 100 μm). (f) The relative invasion (%) was analyzed by Image J and compared to 0.01% PBS control cells. (g) The gene expression of *MMP9* was performed on MDA-MB-231 cells after 24 h of conditioned medium treatments (CM-M0, CM-M1 and CM-M2). Data were collected from triplicate repeats and expressed as mean ± SEM. The significance was compared to control groups by Dunnett's t-test with * $P < 0.05$.

Supplementary Figure 3

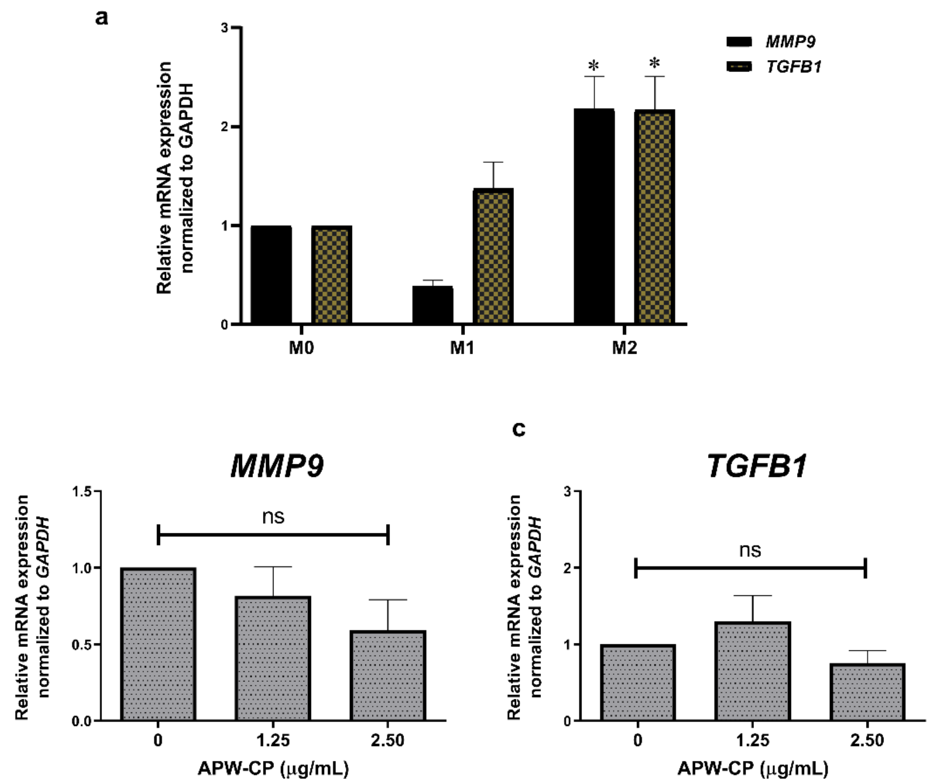

Supplementary Figure 3 The additional M2 phenotype marker determination with M0, M1, M2 and APW-CP exposure on differentiated THP-1 cells. (a) The relative expression of *MMP9* and *TGFB1* genes on M0, M1 and M2 macrophages were analyzed by qRT-PCR and normalized to *GAPDH* as the internal control after 12 h. The determination of (b) *MMP9* and (c) *TGFB1* gene expression with APW-CP treatment (1.25 and 2.5 μg/mL) on macrophages after 12 h and analyzed by qRT-PCR. Data were collected from three biological replicates and expressed as mean ± SEM. Significant results were compared to 0.01% PBS control cells and analyzed by Dunnett's t-test with * $P < 0.05$.

Supplementary Figure 4 Original images for western blot analysis in main figure 4

Original membranes for western blot analysis in Fig.4d. Blots were run for detecting p-p65 NF-κB, Total p65 NF-κB, p-IκBα, Total IκBα and GAPDH proteins. Since p-65 NF-κB (65 kDa) and p-IκBα (40 kDa) exhibit the bands near to each other and p-IκBα showed a noise background. To reduce the non-specific background from re-probing, gels were cut between 48-63 kDa protein markers to detect phosphorylated and total forms of targets. GAPDH, an internal control, were probed to ensure that all these blots were obtained from the same membrane in each replicate. Blue arrows indicate where membranes were cut prior to antibody probing and detection. Images for all replicate blots are shown below.

Fig. 4d

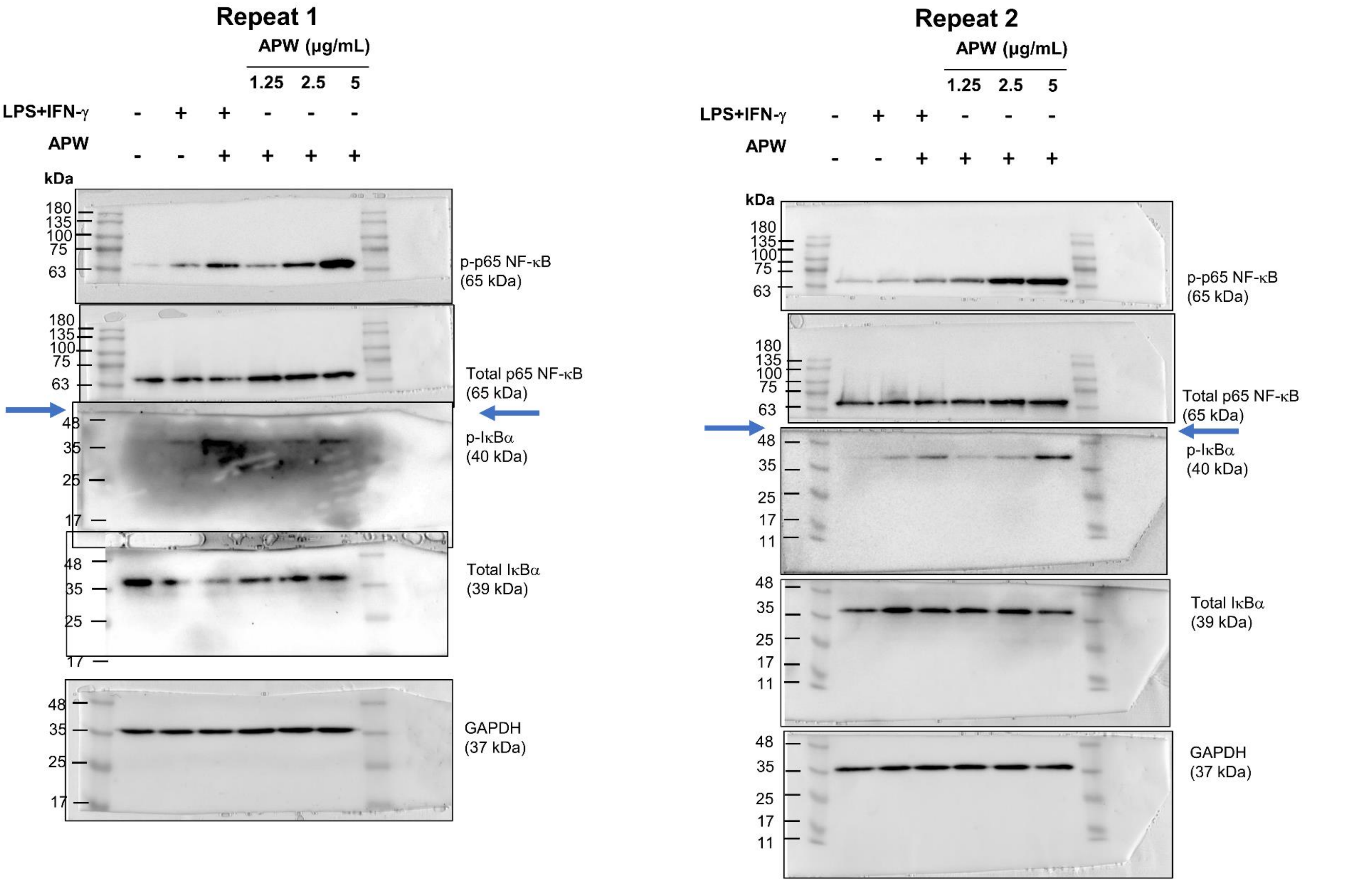

Repeat 1
APW (μg/mL)
1.25 2.5 5
LPS+IFN-γ - + + - - -
APW - - + + + +
kDa
180
135
100
75
63
p-p65 NF-κB (65 kDa)
Total p65 NF-κB (65 kDa)
48
35
25
17
p-IκBα (40 kDa)
Total IκBα (39 kDa)
GAPDH (37 kDa)
Repeat 2
APW (μg/mL)
1.25 2.5 5
LPS+IFN-γ - + + - - -
APW - - + + + +
11

Fig. 4d

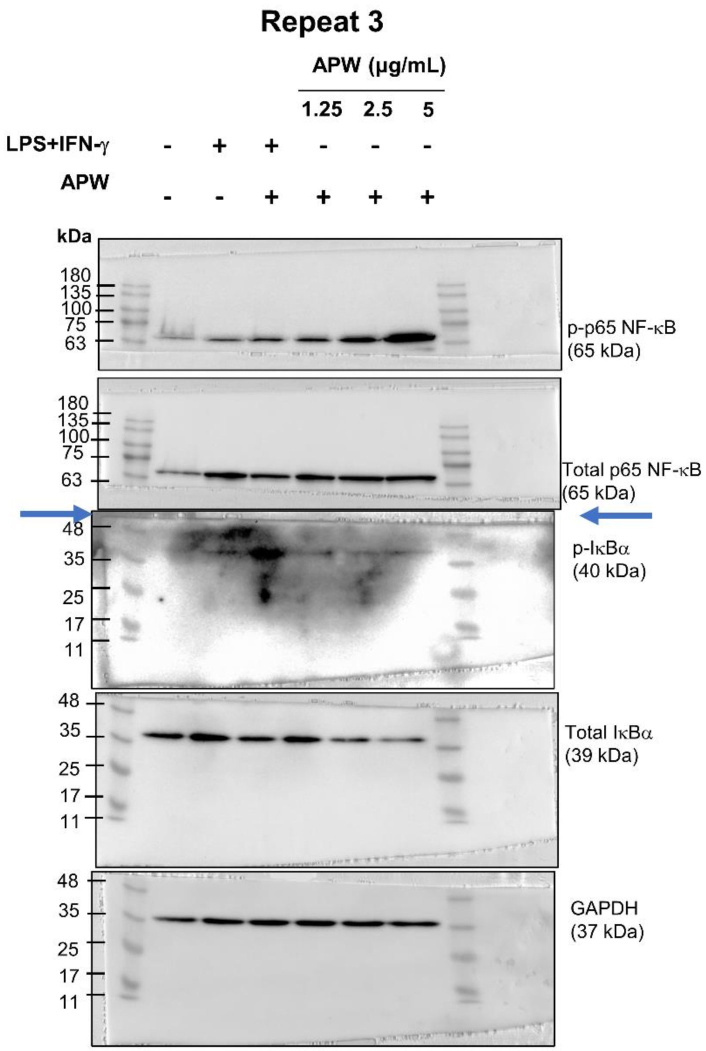

Repeat 3
APW (μg/mL)
1.25 2.5 5
LPS+IFN-γ - + + - - -
APW - - + + + +
kDa
180
135
100
75
63
p-p65 NF-κB (65 kDa)
Total p65 NF-κB (65 kDa)
48
35
25
17
11
p-IκBα (40 kDa)
Total IκBα (39 kDa)
GAPDH (37 kDa)

Fig. 4d

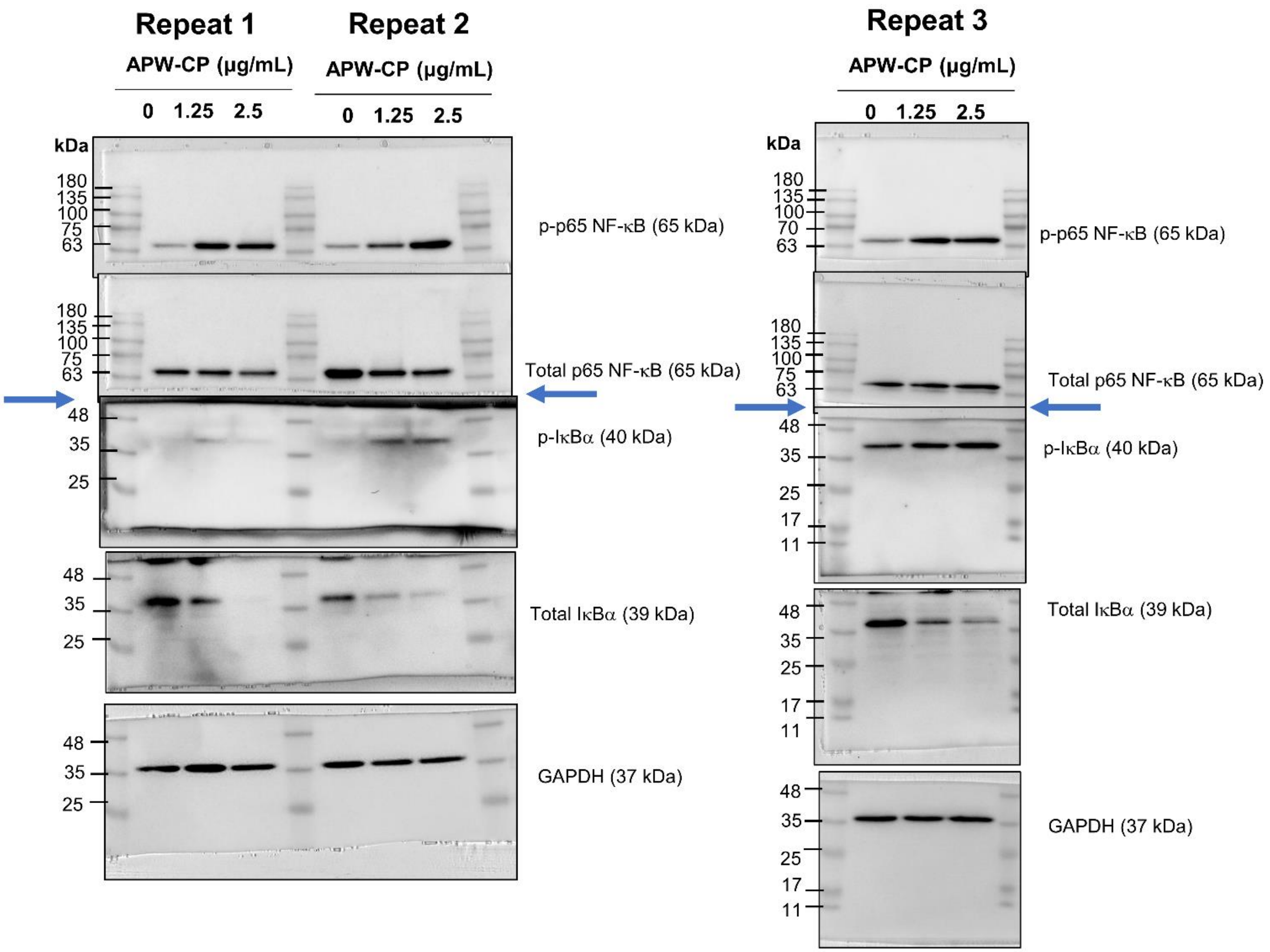

Repeat 1
Repeat 2
Repeat 3
APW-CP (µg/mL)
0 1.25 2.5
kDa
180
135
100
75
70
63
48
35
25
17
11
p-p65 NF-κB (65 kDa)
Total p65 NF-κB (65 kDa)
p-IκBα (40 kDa)
Total IκBα (39 kDa)
GAPDH (37 kDa)
