# Supplementary material for: M1 macrophages polarized by crude polysaccharides isolated from Auricularia polytricha exhibit anti-tumor effect on human breast cancer cells
Source: Sci Rep. 2024 Apr 8;14:8179. doi: 10.1038/s41598-024-58208-2 (PMC11001921; doi:10.1038/s41598-024-58208-2)
Supplement: Supplementary file 2 — Supplementary Tables. [file 41598_2024_58208_MOESM2_ESM.pdf]

Supplementary information

Title

M1 macrophages polarized by crude polysaccharides isolated from *Auricularia polytricha* exhibit anti-tumor effect on human breast cancer cells

Authors

Sunita Nilkhet[1], Kuljira Mongkolpobsin[2,] Chanin Sillapachaiyaporn[3], Nichaporn Wongsirojkul[2], Tewin Tencomnao[3] and Siriporn Chuchawankul[2,4 *]

Affiliations

[1] Program in Clinical Biochemistry and Molecular Medicine, Department of Clinical Chemistry, Faculty of Allied Health Sciences, Chulalongkorn University, Bangkok, 10330, Thailand.

[2] Department of Transfusion Medicine and Clinical Microbiology, Faculty of Allied Health Sciences, Chulalongkorn University, Bangkok 10330, Thailand.

[3] Department of Clinical Chemistry, Faculty of Allied Health Sciences, Chulalongkorn University, Bangkok, 10330, Thailand.

[4] Immunomodulation of Natural Products Research Unit, Chulalongkorn University, Bangkok, 10330, Thailand.

[*] Corresponding author: Siriporn.ch@chula.ac.th (S.C.)

Supplementary tables

Table 1S The primer sequences used in qRT-PCR

Gene	NCBI Reference		Sequence (5'-3')
CD86	NM_175862.5	Forward	CACAGCAGAAGCAGCCAAAATG
		Reverse	CTTCAGAGGAGCAGCACCAGA
CD206	NM_002438.4	Forward	GCCCGGAGTCAGATCACACA
		Reverse	AGTGGCTCAACCCGATATGACAG
IL6	NM_000600.5	Forward	AGTGAGGAACAAGCCAGAGC
		Reverse	AGCTGCGCAGAATGAGATGA
IL10	NM_000572.3	Forward	CAGGGCACCCAGTCTGAGAAC
		Reverse	AGGCTTGGCAACCCAGGTAA
IL1B	NM_000576.3	Forward	CAGAAGTACCTGAGCTCGCC
		Reverse	AGATTCGTAGCTGGATGCCG
TNFA	NM_000594.4	Forward	CCCGAGTGACAAGCCTGTAG
		Reverse	GAGGTACAGGCCCTCTGATG
GAPDH	NM_002046.7	Forward	ATGTTCGTCATGGGTGTGAA
		Reverse	ACAGTCTTCTGGGTGGCAGT
MMP9	NM_004994.3	Forward	TTCGACGATGACGAGTTGTG
		Reverse	TCGAAGATGAAGGGGAAGTG
TGFB1	NM_000660.7	Forward	CCCTGGACACCAACTATTGC
		Reverse	GTCCAGGCTCCAAATGTAGG
